# Preparation and Performance Evaluation of Graphene Oxide-Based Self-Healing Gel for Lost Circulation Control

**DOI:** 10.3390/polym17151999

**Published:** 2025-07-22

**Authors:** Wenzhe Li, Pingya Luo, Xudong Wang

**Affiliations:** 1 Petroleum Engineering School, Southwest Petroleum University, Chengdu 610500, China; 2 Engineering Technology Research Institute, PetroChina Southwest Oil & Gas Field Company, Chengdu 610017, China

**Keywords:** drilling fluid, self-healing gel, nanocomposite gels, lost circulation control

## Abstract

Lost circulation is a major challenge in oil and gas drilling operations, severely restricting drilling efficiency and compromising operational safety. Conventional bridging and plugging materials rely on precise particle-to-fracture size matching, resulting in low success rates. Self-healing gels penetrate loss zones as discrete particles that progressively swell, accumulate, and self-repair in integrated gel masses to effectively seal fracture networks. Self-healing gels effectively overcome the shortcomings of traditional bridging agents including poor adaptability to fractures, uncontrollable gel formation of conventional downhole crosslinking gels, and the low strength of conventional pre-crosslinked gels. This work employs stearyl methacrylate (SMA) as a hydrophobic monomer, acrylamide (AM) and acrylic acid (AA) as hydrophilic monomers, and graphene oxide (GO) as an inorganic dopant to develop a GO-based self-healing organic–inorganic hybrid plugging material (SG gel). The results demonstrate that the incorporation of GO significantly enhances the material’s mechanical and rheological properties, with the SG-1.5 gel exhibiting a rheological strength of 3750 Pa and a tensile fracture stress of 27.1 kPa. GO enhances the crosslinking density of the gel network through physical crosslinking interactions, thereby improving thermal stability and reducing the swelling ratio of the gel. Under conditions of 120 °C and 6 MPa, SG-1.5 gel demonstrated a fluid loss volume of only 34.6 mL in 60–80-mesh sand bed tests. This gel achieves self-healing within fractures through dynamic hydrophobic associations and GO-enabled physical crosslinking interactions, forming a compact plugging layer. It provides an efficient solution for lost circulation control in drilling fluids.

## 1. Introduction

Drilling engineering is an important part of oil and gas exploitation, and lost circulation is a common complex situation in drilling engineering. Lost circulation mainly refers to the phenomenon in which the drilling fluid leaks into the formation through the borehole wall under the action of a downhole pressure difference during downhole operation. Lost circulation not only leads to substantial drilling fluid depletion but also triggers wellbore instability, potentially inducing cascading downhole complications such as blowouts, cave-ins, and related anomalies. These issues collectively escalate non-productive time (NPT), prolong drilling cycles, amplify operational complexity and costs, and severely impede the exploration and development of hydrocarbon resources [1,2,3]. Therefore, lost circulation has become the primary major problem restricting the quality and efficiency of oil and gas drilling engineering [4,5].

With the continuous advancement of hydrocarbon exploration and development, drilled formations are becoming increasingly complex. The plugging effectiveness of currently used bridging and plugging materials heavily relies on the matching degree between particle size and lost circulation channel dimensions, resulting in low plugging success rates and high susceptibility to further loss [6,7]. Self-healing-gel plugging materials have reversible dynamic bonds and deformability. Therefore, self-healing gel not only exhibits self-adaptability to fracture dimensions but also effectively enhances the interaction forces between plugging particles, thereby increasing the strength of the plugging layer [8,9,10]. Self-healing gels effectively overcome the shortcomings of traditional bridging agents including poor adaptability to fractures, uncontrollable gel formation of conventional downhole crosslinking gels, and the low strength of conventional pre-crosslinked gels. However, currently reported self-healing gels exhibit excessively rapid water absorption rates in working fluids due to their hydrophilicity and swellability, while demonstrating poor adhesion and stability within formations [10]. Nanocomposite gel refers to the inorganic nano/polymer composite gel obtained by compounding inorganic nanoparticles with hydrogel [11,12,13]. During the preparation process, nanomaterials are primarily dispersed through mechanical stirring, ultrasonic dispersion, or surface modification methods. Subsequently, monomer polymerization occurs within the highly dispersed nano-solution to yield nanocomposite gels [14,15,16]. Nanocomposite gel not only has thermal stability and strength, but also retains the advantages of deformability, processability, and water absorption of hydrogel, which makes up for the shortcomings in poor mechanical strength of traditional gel [17,18,19]. GO is a two-dimensional nanomaterial with monoatomic thickness, produced by oxidizing natural graphite. GO exhibits properties including low density, a large specific surface area, and abundant oxygen-containing functional groups on its surface. It can be incorporated into hydrogel structures through various supramolecular interactions such as hydrogen bonding, van der Waals forces, electrostatic interactions, and π-π stacking. Owing to its large specific surface area and hydrophilic polar groups, GO is expected to significantly enhance the mechanical strength, stability, and other properties of polymeric hydrogels. It is therefore imperative to develop a high-performance self-healing nanocomposite gel with tailored adhesive properties and controlled swelling kinetics for drilling fluid loss control.

In this paper, stearyl methacrylate (SMA) was used as a hydrophobic monomer and acrylamide (AM) and acrylic acid (AA) were used as hydrophilic monomers to form a hydrophobic-association polymer with self-healing properties by free radical polymerization. Then GO was introduced to form an SG self-healing gel with the hydrophobic-association polymer. The self-healing properties, swelling behavior, and mechanical, adhesion, and plugging properties of SG gel were systematically tested, and the physical and chemical properties of SG gel were characterized by XRD, FT-IR, SEM, TGA, and Raman spectroscopy. SG gel provides a new lost circulation material (LCM) for drilling fluid loss control.

## 2. Experimental Section

### 2.1. Materials

Flake graphite (99%) was purchased from Beijing InnoChem Science & Technology Co., Ltd. (Beijing, China) Acrylamide (AM, 99%), stearyl methylacrylate (SMA, 96%), sodium dodecyl sulfate (SDS, 97%), acrylic acid (AA, 99%), sodium chloride (NaCl, 99.5%), calcium chloride (CaCl_2_), and ammonium persulphate (APS, 98.5%) were obtained from Aladdin, Shanghai, China. Sulfuric Acid (H_2_SO_4_), sodium Nitrate (NaNO_3_), potassium permanganate (KMnO_4_), hydrogen peroxide (H_2_O_2_), hydrochloric acid (HCl), and ethanol were purchased from Chengdu Chron Chemicals Co., Ltd. (Chengdu, China).

### 2.2. Preparation Method

(1) Preparation of GO

GO was prepared according to a method in the literature [20]. Specifically, 9 g of flake graphite was added to H_2_SO_4_ (270 mL), followed by the slow addition of 4.5 g NaNO_3_ powder with continuous stirring for 30 min. Subsequently, KMnO_4_ (27 g) was gradually introduced into the mixture under continuous agitation for 12 h. The mixture was then stirred at 90 °C for 20 min, and 1200 mL deionized water was slowly added. After cooling, 50 mL H_2_O_2_ was added dropwise, and the solution was left to stand for 12 h. The resulting suspension was centrifuged, washed with HCl and ethanol, and freeze-dried to obtain GO powder.

(2) Preparation of SG gel

**Figure 1:**
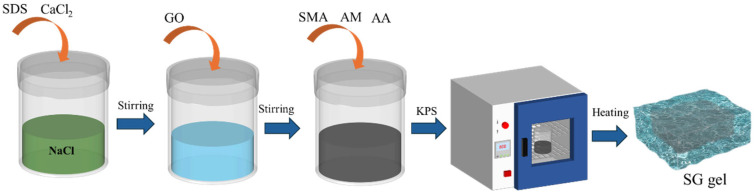
The preparation process of SG gel.

As shown in Figure 1, 0.1 g CaCl_2_ and 2 g SDS were added to 0.6 mol/L NaCl solution (30 mL), and a certain amount of GO (0, 0.5wt%, 1.5wt%) was slowly added at 35 °C under magnetic stirring to obtain suspension A. Then, 0.3 g SMA, 4 g AM, and 1 g AA were added to the above-mentioned suspension A, and magnetic stirring was continued at the same temperature for 4 h to form the uniform black suspension B. Finally, 0.05 g APS was added to the above-mentioned suspension B and stirred evenly [8,21]. The suspension was then placed in an oven at 50 °C for 8 h to obtain SG-0, SG-0.5, and SG-1.5 gels, where SG-0, SG-0.5, and SG-1.5 represent gels with different GO addition quantities.

### 2.3. Characterization

The synthesized samples were characterized by SEM, Raman spectroscopy, FT-IR, TGA, and XRD. The characterization is detailed in the Supplementary Materials.

### 2.4. Mechanical and Rheological Property Tests

The rheological test of the self-healing gel was carried out by a rotary rheometer (MCR 320e, Anton Paar, Graz, Austria), and the tensile test of the self-healing gel was carried out by an electronic universal material testing machine (WH-50, Weiheng, Ningbo, China). The detailed methods are described in the Supplementary Materials.

### 2.5. Self-Healing and Expansion Performance Tests

The self-healing time of the sample was determined by a bottle test [10]. The dried gel particles were added to a plugging base mud (5% mud) to prepare a dispersion with a gel particle concentration of 2%. After stirring evenly, the sample was placed in an oven at different temperatures, and the morphology of the sample was checked every 0.5 h. In this paper, the self-healing time is defined as the time when the boundary between the gels disappears.

The gel particles with initial mass M_0_ were placed in an excessive plugging base mud (5% mud) and fully swelled at different temperatures [8,10]. The mass M_t_ of the gel was recorded at intervals, and the swelling ratio (SR) of the sample was determined by Equation (1).

(1) $$SR=\frac{M_{t}-M_{0}}{M_{0}}$$

where M_0_ and M_t_ are the mass of the sample at the initial time and time t, respectively.

### 2.6. Self-Healing and Expansion Performance Tests

First, 100 g of quartz sand was weighed and placed at the bottom of a drilling fluid cell to simulate a loss zone. Then, 400 mL of prepared plugging slurry was added to the mud cell. After the temperature was raised to the set value and stabilized, the upper and lower valves were opened. The pressure differential between the valves was increased to 6 MPa at a ramp rate of 1 MPa/2 min, during which the lost circulation volume V_1_ was measured. Subsequently, the valves were closed, and the pressure in the mud cell was reduced to 1 MPa. After 6 h, the valves were reopened, and the pressure differential was again increased to 6 MPa at the same ramp rate (1 MPa/2 min), with the lost circulation volume V_2_ recorded. The cumulative lost circulation volume was calculated as V = V_1_ + V_2_.

## 3. Results and Discussion

### 3.1. Synthesis of SG Gel

**Figure 2:**
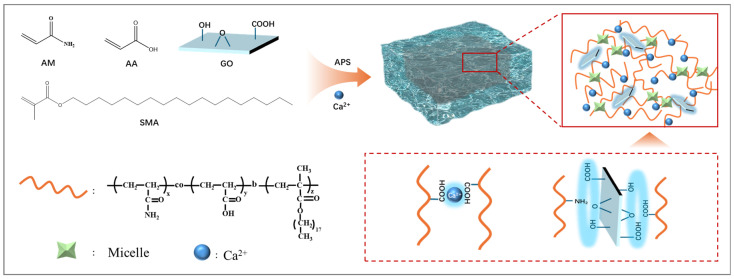
Formation mechanism of the SG self-healing gel.

The SG self-healing gel prepared in this study is primarily based on intermolecular hydrophobic interactions, metal coordination interactions, and hydrogen bonding, with its formation process illustrated in Figure 2. Initially, sodium dodecyl sulfate (SDS) forms micelles in a NaCl solution. The initiator ammonium persulfate (APS) decomposes under heating to generate free radicals, which attack acrylamide (AM), acrylic acid (AA), and stearyl methacrylate (SMA) monomer molecules to form monomeric free radicals. Consequently, the hydrophilic monomers (AM and AA) copolymerize with the hydrophobic monomer (SMA) to generate micelle-connected polymers. Furthermore, after the SMA monomer is solubilized within SDS micelles, it polymerizes to form P(SMA), serving as a dynamic crosslinking point for hydrophobic association. Concurrently, carboxyl groups (COOH) on the polymer molecular chains coordinate with free Ca^2+^ ions, which enhances the crosslinking degree of the gel [22,23,24]. The abundant hydroxyl, carboxyl, and epoxy groups on the surface of GO can form hydrogen-bonded physical crosslinks with the amino and carboxyl groups of P(SMA-AM-AA), thereby further strengthening the intermolecular interactions and enhancing the mechanical properties of the SG gel.

### 3.2. Characterization of SG Gel

**Figure 3:**
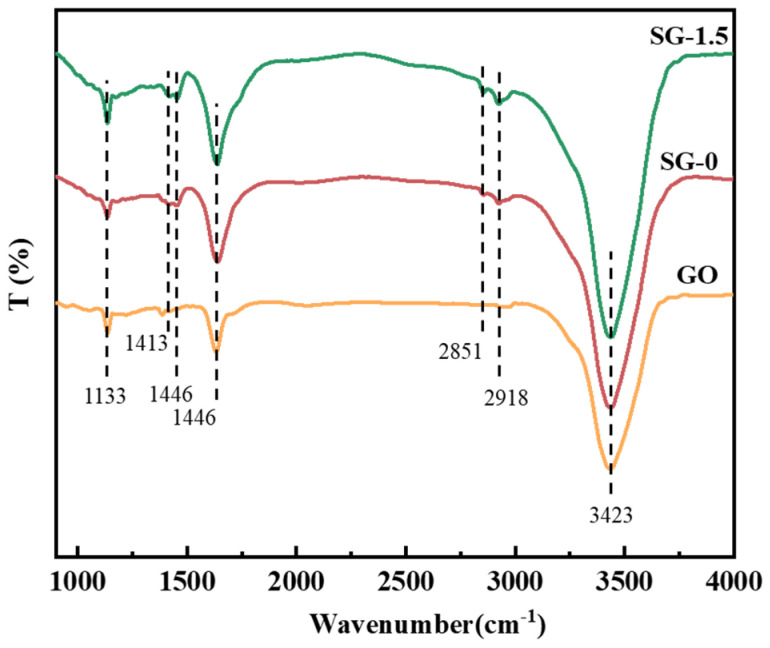
FT-IR absorption peaks of the samples.

To obtain the functional group information of the samples, freeze-dried SG-0 and SG-1.5 gels and GO were subjected to FT-IR characterization, with the results shown in Figure 3. All samples exhibit distinct absorption peaks at 3423 cm^−1^, 1631 cm^−1^, and 1133 cm^−1^. The broad peak near 3423 cm^−1^ corresponds to the O–H stretching vibration, the characteristic peak around 1631 cm^−1^ is attributed to the C=O stretching vibration of COO^−^ groups, and the strong absorption peak at 1133 cm^−1^ arises from the C–O stretching vibration [25,26]. Furthermore, the SG-1.5 gel demonstrates higher intensities of the characteristic peaks at 3423 cm^−1^, 1631 cm^−1^, and 1133 cm^−1^ compared to SG-0, which may result from the superposition of characteristic peaks induced by the incorporation of GO. For the SG-0 and SG-1.5 gels, the absorption peaks at 2918 cm^−1^ and 2851 cm^−1^ are attributed to the C–H stretching vibrations in the molecular chains, while the peaks at 1413 cm^−1^ and 1446 cm^−1^ correspond to the bending vibrations of methyl groups. The above results demonstrate the presence of –OH, –COOH, and epoxy groups on the surface of GO, and confirm that no new functional groups were introduced during the synthesis of the SG-1.5 gel.

**Figure 4:**
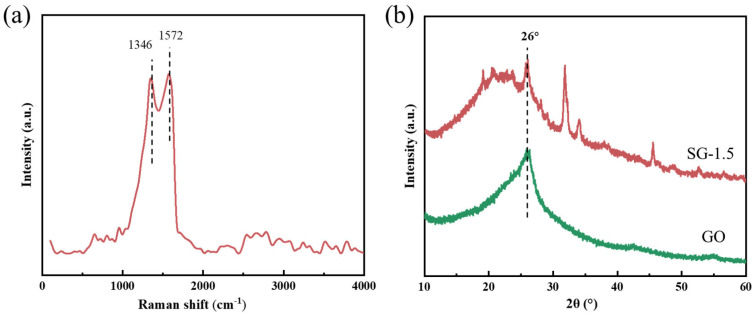
Raman and XRD characterization of the samples: (**a**) Raman spectrum of the SG-1.5 gel; (**b**) XRD patterns of the SG-1.5 gel and GO.

Raman spectroscopy is commonly employed to characterize the structural features of carbon materials. To further confirm the successful incorporation of GO into the SG gel, Raman characterization was conducted on the SG-1.5 gel (Figure 4a). Prominent characteristic peaks were observed at approximately 1346 cm^−1^ and 1572 cm^−1^, corresponding to the D band and G band of carbon materials, respectively, indicating the typical amorphous nature of GO [27,28,29]. This confirms the successful incorporation of GO into the SG-1.5 gel. XRD characterization was performed to analyze the crystalline structures of the SG-1.5 gel and GO, with the results shown in Figure 4b. A distinct characteristic diffraction peak for GO was observed near 2θ ≈ 26.26°, corresponding to the (002) crystal plane of graphene [30]. Moreover, the XRD pattern of SG-1.5 gel also showed the same characteristic peak near 2θ ≈ 26°, which further indicated that GO was doped into the self-healing gel.

**Figure 5:**
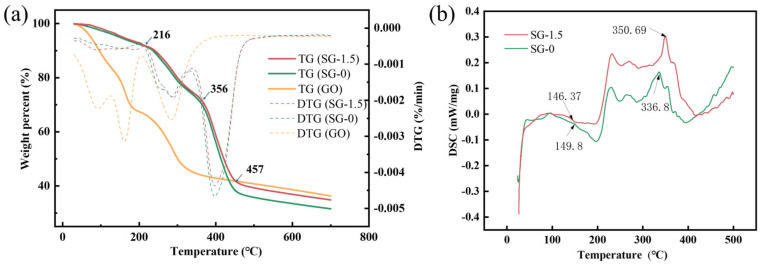
Thermal stability analysis of the samples: (**a**) TG-DTG curves; (**b**) DSC curves.

Thermal stability is a critical indicator for evaluating plugging materials. Figure 5 presents the TG and DTG curves of GO and the SG-0 and SG-1.5 gels under a nitrogen atmosphere. The TG curves of both the SG-0 and SG-1.5 gels exhibit identical mass loss trends, and their DTG curves show two distinct decomposition peaks. The mass loss process can be divided into the following three stages: For the SG-1.5 gel, the mass loss of all samples below 216 °C is relatively minor, which is primarily attributed to the evaporation of bound water and free water within the samples. The mass loss in the temperature range of approximately 216 °C to 356 °C is primarily caused by the decomposition of carboxyl groups on the polymer molecular chains, the pyrolysis of two adjacent amino groups on the side chains forming imine structures, and the degradation of oxygen-containing functional groups in GO. When the temperature exceeds 356 °C, the polymer skeleton collapses, the three-dimensional structure becomes disrupted, and GO initiates thermal decomposition [28,31]. In addition, the Tg and Tm of the samples were determined by DSC tests, and the results are shown in Figure 1. The Tg and Tm values of SG-0 were 149.8 °C and 336.8 °C, respectively, and the Tg and Tm values of SG-1.5 were 146.37 °C and 350.69 °C, respectively (Figure 5b). This shows that the introduction of GO has little effect on the Tg of the gel, but the melting temperature increases. The findings conclusively demonstrate that the SG gel exhibits exceptional thermal stability, with the incorporation of GO showing no measurable impact on the material’s temperature resistance characteristics.

**Figure 6:**
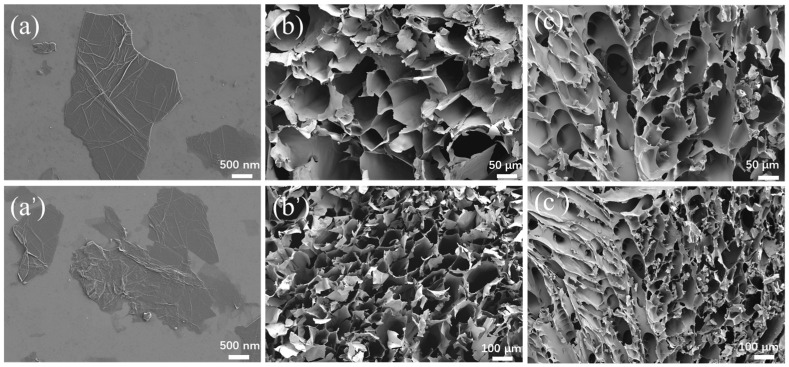
SEM images of the samples: (**a**,**a’**) GO; (**b**,**b’**) SG-0; (**c**,**c’**) SG-1.5.

**Figure 7:**
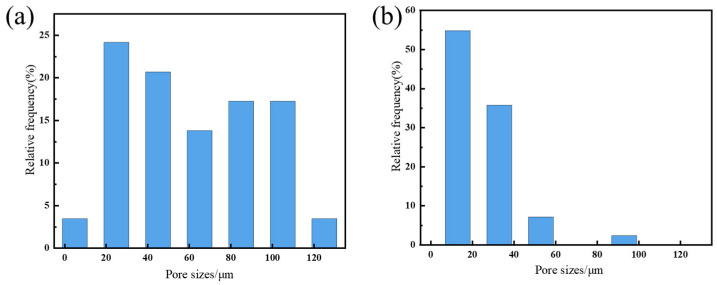
Pore size distribution of the samples: (**a**) SG-0; (**b**) SG-1.5.

The microstructures of GO and the SG-0 and SG-1.5 gels were characterized by SEM, with the results shown in Figure 6. The synthesized graphene oxide exhibits a dispersed lamellar structure with surface wrinkling, confirming successful preparation of the graphene oxide. Both the SG-0 and SG-1.5 gels demonstrate a characteristic three-dimensional network architecture, which arises from the hydrophobic association of long alkyl chains in the hydrophobic monomers forming dynamic crosslinking sites, while coordination interactions between Ca^2+^ ions and carboxyl groups further enhance the crosslinking density of the gel system [32]. Additionally, the pore sizes of SG-0 are predominantly distributed between 20 μm and 100 μm, while those of SG-1.5 primarily fall within the 20–40 μm range (Figure 7). This indicates that after GO incorporation, the pore structure within the SG-1.5 hydrogel network progressively diminishes, resulting in an increasingly compact architecture. This phenomenon is attributed to crosslinking interactions between the polymer and GO. Due to abundant hydroxyl, carboxyl, and epoxy functional groups on the GO surface, hydrogen-bonded physical crosslinks form with amino and carboxyl groups in the P(SMA-AM-AA) polymer chains. This enhancement of intermolecular forces effectively increases the crosslinking density within the hydrogel network, further supporting the existence of molecular-level interactions between GO and polymer chains.

### 3.3. Performance Evaluation of SG Gels

(1) Swelling properties

**Figure 8:**
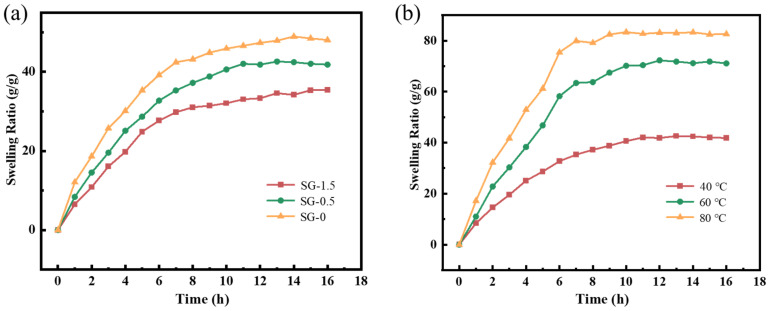
Swelling performance of the samples: (**a**) swelling behavior of different samples; (**b**) swelling behavior under different temperatures.

Figure 8a demonstrates the swelling behavior of SG-1.5 gel particles in bentonite slurry. During the initial swelling stage of all gel particles, the swelling ratio exhibited a rapid increase followed by a gradual decline. This phenomenon is primarily attributed to the reorganization of dynamic bonds within the gel during swelling, which resulted in the increased crosslinking density and improved structural stability of the network architecture. The equilibrium swelling ratios of SG-0, SG-0.5, and SG-1.5 gel particles were measured to be 48.9 g/g, 42.3 g/g, and 34.1 g/g, respectively, demonstrating an inverse correlation between GO content and swelling capacity. This phenomenon is attributed to interfacial interactions between GO and polymer chains, where increased GO loading enhances crosslinking density and generates a more compact network architecture, thereby restricting water molecule diffusion through the gel matrix [8,17]. The swelling behavior of gels at different temperatures was investigated using SG-1.5 as an example, with the results shown in Figure 8b. The equilibrium swelling ratios of SG-1 gel particles at 40 °C, 60 °C, and 80 °C were measured to be 42.6 g/g, 72.6 g/g, and 83.5 g/g, respectively. As the temperature increased, the equilibrium swelling ratio of SG-1.5 gel particles gradually increased while the time required to reach equilibrium swelling decreased. This phenomenon is primarily attributed to the accelerated diffusion of water molecules at elevated temperatures.

(2) Rheological properties

**Figure 9:**
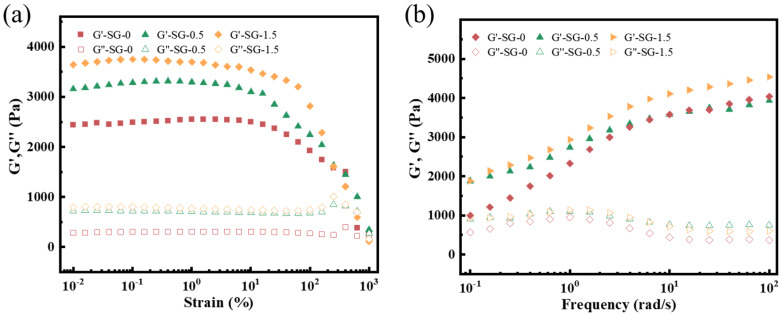
Rheological properties of the samples: (**a**) strain scanning curves; (**b**) frequency scanning curves.

Rheological strength is a critical performance parameter of plugging materials. Figure 9a demonstrates the rheological property test results of SG gel. Within the linear viscoelastic region, the storage modulus (G′) values of all gels exceeded their corresponding loss modulus (G″) values, indicating their solid-like behavior [33]. SG-0 exhibited a lower G′ of merely 2524 Pa, while SG-0.5 and SG-1.5 demonstrated G′ values of 3299 Pa and 3750 Pa, respectively. This indicates that the G′ values of SG gels progressively increase with elevated GO content. This phenomenon arises because incorporating higher amounts of the inorganic nanomaterial GO enhances the crosslinking density of the composite, which intensifies intermolecular interactions within the gel network, thereby improving the material’s rheological strength [8]. The frequency sweep results of the post-self-healing gel within the 0.1~100 rad/s range are shown in Figure 9b. The gel samples consistently exhibited G′ values surpassing those of G′′ across the entire frequency range. Meanwhile, a gradual increase in G′ values was observed with ascending frequency, indicating that the gel retained elastic characteristics and demonstrated frequency-dependent behavior in G′. This phenomenon arises from dynamic crosslinking interactions within the SG gel structure, including hydrophobic associations, coordination interactions, and hydrogen bonds, which render the modulus variations frequency-responsive [34].

(3) Mechanical properties

**Figure 10:**
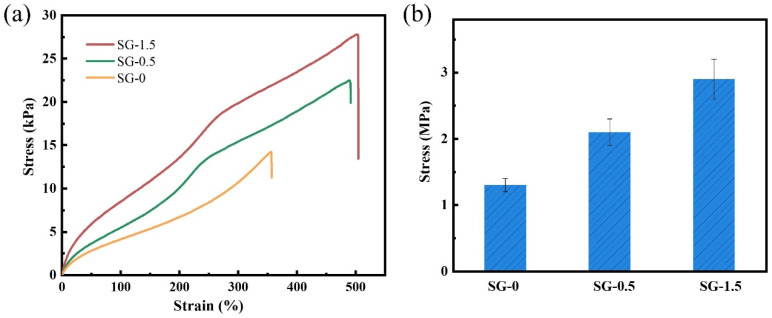
Mechanical properties of the samples: (**a**) tensile properties; (**b**) compressive properties.

The tensile properties of the gels were tested using a universal testing machine, with the results presented in Figure 10a. The SG-0 gel exhibited an elongation at break of 356% with a tensile fracture stress of merely 13.2 kPa. The SG-0.5 gel demonstrated values of 487% for elongation at break and 22.3 kPa for tensile fracture stress. With an increasing GO content, the tensile strength showed progressive enhancement, culminating in the SG-1.5 gel achieving 504% elongation at break and 27.1 kPa tensile fracture stress. A parallel trend was observed in the compressive strength of the self-healing gels (Figure 10b). The unique lamellar structure of GO facilitates energy dissipation through localized sliding between adjacent layers, thereby preventing relative displacement and fracture motion of molecular chains within the gel network and consequently enhancing the gel’s mechanical strength. Furthermore, the tight integration between GO and the hydrophobic-associative polymer matrix enables the formation of non-covalent interactions, including ionic bonds and hydrogen bonds, between the oxygen-containing functional groups on GO sheets and polymer chains, as well as among adjacent polymer molecular chains. These interactions act as energy dissipation sites during tensile or compressive deformation processes. With the increase in GO content, the number of internal hydrogen bonds gradually increased, and the density of the internal crosslinking network gradually increased, thus improving the mechanical strength of the composite gel and maintaining a stable structure under external stress.

(4) Adhesion properties

**Figure 11:**
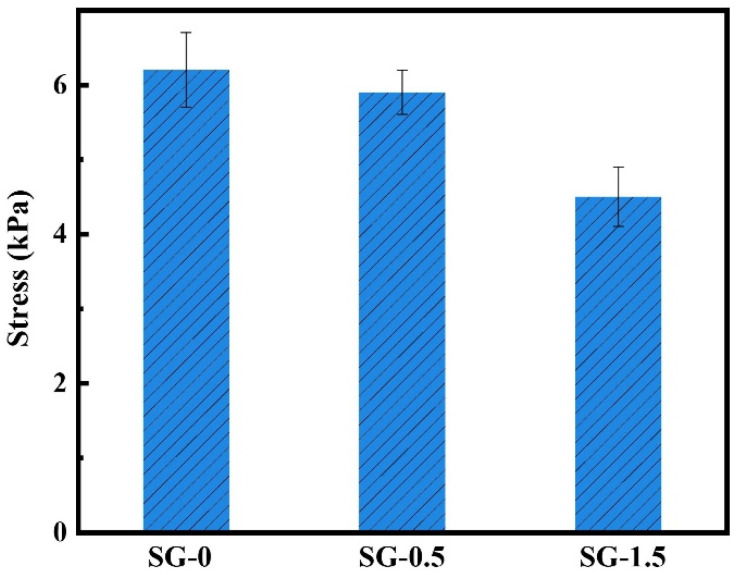
Adhesion properties of samples.

Adhesive gels mainly adhere to other objects through hydrogen bonds, host–guest interactions, metal–ligand complexation interactions, and ion interactions. SG gel also showed adhesion properties, and the adhesion strength of SG-0 and SG-0.5 was comparable (Figure 11). When the GO content was high, the adhesion performance of SG-1.5 gel was significantly reduced. This further indicates that there is an interaction between GO and the polymer molecular chain. GO oxygen-containing groups have hydrogen bonding interactions with the carboxyl groups of the polymer molecular chain, resulting in a decrease in the number of active carboxyl groups of the composite gel that can bind to the tissue, so the adhesion strength is reduced [35].

(5) Self-healing properties

**Figure 12:**
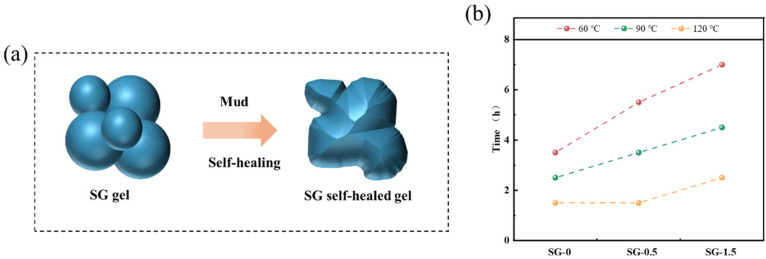
Self-healing properties of the sample: (**a**) self-healing process; (**b**) self-healing time.

Due to the presence of dynamic reversible bonds in the SG gel structure, the dried SG gel particles can self-heal after swelling in bentonite mud. The self-healing time can be determined by observing the duration required for the swollen SG gel particles to integrate into a monolithic structure (Figure 12a). The self-healing times of different SG gels exhibited variations. SG-0 required 3.5 h, 2.5 h, and 1.5 h for self-healing at 60 °C, 90 °C, and 120 °C, respectively (Figure 12b). The self-healing durations were further prolonged for SG-0.5 and SG-1.5, requiring 5.5 h and 7 h, respectively, at 60 °C to complete the self-repair process. This demonstrates that the self-healing time of SG gels prolongs with a higher GO content. This phenomenon can be attributed to the inherent characteristics of GO as a typical inorganic nanomaterial. With an increasing GO concentration, the mobility of polymer chains becomes constrained by the inorganic GO components, thereby reducing self-healing efficiency [36,37]. The observed reduction in self-healing time at elevated temperatures results from enhanced polymer chain mobility induced by thermal activation, which facilitates the self-healing process.

(6) Plugging performance

To meet the operational requirements of gel plugging, dry SG-1.5 gel particles were prepared into a 4%-concentration-gel plugging slurry. Tests conducted with a six-speed rotational viscometer revealed that SG-1.5 gel increased both the AV and PV of the bentonite slurry. Additionally, it enhanced dynamic shear force and reduced medium-pressure fluid loss to some extent (Table 1). These results indicate that the SG gel, after water absorption and swelling in the bentonite slurry, can strengthen the gel structure of the soil slurry through enhanced crosslinking interactions between colloidal particles.

**Figure 13:**
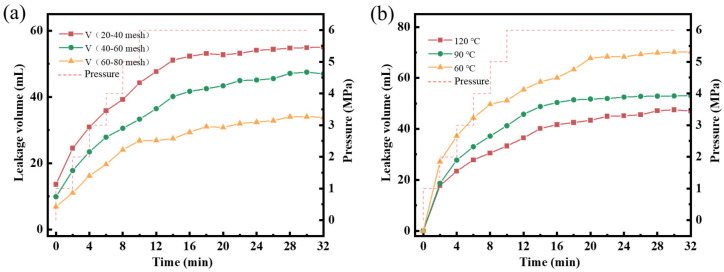
Plugging performance of SG-1.5 gel: (**a**) plugging performance of SG-1.5 gel for sand beds with different particle sizes; (**b**) plugging performance of SG-1.5 gel at different temperatures.

The plugging performance of SG-1.5 gel was evaluated using sand beds with 20–40-mesh, 40–60-mesh, and 60–80-mesh particle sizes (Figure 13). The SG-1.5 gel demonstrated cumulative leakage volumes of 54.3 mL (20–40 mesh), 45.7 mL (40–60 mesh), and 34.6 mL (60–80 mesh) under conditions of 6 MPa pressure and 120 °C temperature over 30 min. These results indicate that the gel has an excellent plugging effect and significantly reduces fluid leakage. This is attributed to the dynamic reversible structure of SG-1.5 gel. Under the high-temperature excitation of the formation, the hydrophobic group of SG-1.5 gel is reorganized, and Ca^2+^ is re-coordinated with free carboxylate to achieve ion crosslinking. Concurrently, GO forms a reversible non-covalent bond with the functional group of the polymer molecular chain, thus completing the dynamic repair of the structure and then forming a stable plugging layer in the fracture. Additionally, SG-1.5 gel demonstrates effective plugging performance on three different sizes of sand beds, indicating that SG-1.5 gel exhibits excellent self-adaptability to fractures of varying dimensions. The SG-1.5 gel can enter fractures to form plugging layers and reduce fluid loss. Meanwhile, the cumulative fluid loss volumes of SG-1.5 gel at 60 °C and 90 °C over 30 min were 69.9 mL and 52.7 mL, respectively, demonstrating superior plugging performance at 90 °C compared to 60 °C. This is because the SG-1.5 gel has a higher degree of self-healing at 90 °C, the force between the gel particles is stronger, and the formed plugging layer is more complete and denser.

### 3.4. Plugging Mechanism of SG Gels

**Figure 14:**
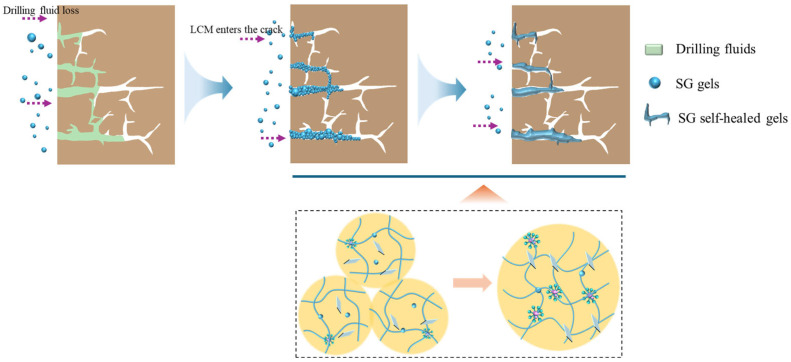
Plugging mechanism of self-healing gel.

Based on the self-healing properties and molecular structure of SG gel, its leakage plugging mechanism in formation fracture plugging can be inferred as follows: The water absorption rate of SG gel in a bentonite slurry can be regulated by controlling the addition amount of GO (Figure 14). Upon absorbing water from the bentonite slurry, the SG gel forms a deformable gel with shape-adaptive capacity. Subsequently, the deformable SG gel enters leakage channels through deformation and differential pressure [38]. By adhering to fracture surfaces and undergoing interparticle stacking, the preliminary plugging layer is initially formed [39]. Throughout this process, the SG gel continues to absorb water and progressively increases in volume. With prolonged time and thermal activation, the polymer molecular chains between adjacent yet discrete SG gel units exhibit mobility. Concurrently, GO and Ca^2+^ undergo interfacial interdiffusion and migration, while free P(LMA) segments become resolubilized by SDS micelles [10]. SG gel molecules gradually reorganize through hydrophobic association, coordination, and non-covalent bonds between GO and polymers, ultimately self-healing from multiple independent gel segments into a whole integrated gel. This significantly enhances the compactness of the plugging layer, effectively plugging leakage channels and reducing fluid loss volume.

## 4. Conclusions

This work successfully prepared a GO-reinforced organic–inorganic self-healing plugging gel (SG gel). The incorporation of GO strengthened the gel network structure through physical crosslinking, leading to enhancements in the mechanical strength and rheological performance of the SG-1.5 gel. The swelling rate of SG gels could be effectively controlled by adjusting the GO content. The equilibrium swelling ratios for SG-0, SG-0.5, and SG-1.5 were measured to be 48.9 g/g, 42.3 g/g, and 34.1 g/g, respectively. The SG-1.5 gel demonstrated a G′ of 3299 Pa and tensile stress of 27.1 kPa, with a self-healing time of 1.5 h at 120 °C. Under conditions of 6 MPa pressure and 120 °C temperature, the SG-1.5 gel exhibited a cumulative leakage volume of 34.6 mL (60–80 mesh) within 30 min. Through synergistic reconfiguration driven by dynamic hydrophobic association, Ca^2+^ coordination, and GO interfacial interactions, the gel undergoes self-repair from multiple discrete segments into a unified monolith within fractures under formation temperature, ultimately establishing a reinforced sealing barrier. This LCM integrates both self-adaptive functionality and inherent self-healing capacity, offering a strategy for mitigating drilling fluid loss in complex formations.

## Figures and Tables

**Table 1:** Effect of SG-1.5 gel on rheology and filtration of mud.

	*AV* (mPa·s)	*PV* (mPa·s)	*YP* (Pa)	*FL_API_ *(mL)
Mud	13	8	5	28.5
Mud + 4% SG-1.5	23.5	14	9.5	12.5

## Data Availability

The original contributions presented in this study are included in the article/Supplementary Material. Further inquiries can be directed to the corresponding authors.

## Supplementary Materials

The following supporting information can be downloaded at: https://www.mdpi.com/article/10.3390/polym17151999/s1. Section S1: Characterization methods; Section S2: Mechanical and rheological property tests.
