# Supplementary material for: Preparation and Performance Evaluation of Graphene Oxide-Based Self-Healing Gel for Lost Circulation Control
Source: Polymers (Basel). 2025 Jul 22;17(15):1999. doi: 10.3390/polym17151999 (PMC12349317; doi:10.3390/polym17151999)
Supplement: Supplementary file 1 [file polymers-17-01999-s001.zip › polymers-3676899-supplementary.pdf]

Preparation and performance evaluation of a graphene oxide-based self-healing gel for lost circulation control

Wenzhe Li[1,2], Pingya Luo[1], Xudong Wang[2]*

[1] Petroleum Engineering School, Southwest Petroleum University, Chengdu 610500, China

[2] Engineering Technology Research Institute, PetroChina Southwest Oil & Gas field Company, Chengdu 610017, China

*Correspondence: wangxudong2018@petrochina.com.cn

Section S1. Characterization methods

The morphology of the samples was obtained by scanning electron microscopy (ZEISS Sigma 360). The functional group information of the sample was obtained by Fourier transform infrared spectrometer (Thermo Fisher Scientific Nicolet iS20). The Raman spectrum of the sample was obtained using a Raman spectrometer (Horiba LabRAM HR Evolution) with a laser radiation wavelength of 532 nm. The thermal decomposition information of the sample was obtained using a thermogravimetric analyzer (Mettler TGA/DSC1). The sample was heated from 25 °C to 800 °C at a rate of 10 °C/min in a nitrogen atmosphere. The XRD diffraction patterns of the materials were obtained by X-ray diffractometer (Rigaku Ultima IV).

Section S2. Mechanical and rheological property tests

The rheological test of the self-healing gel was carried out by a rotary rheometer (MCR 320e, Anton Paar), and the plate spacing was 1 mm. The fixed frequency of strain scanning was 1 Hz, and the strain amplitude scanning range was 0.1% ~ 1000%. The tensile test of the self-healing gel was carried out by an electronic universal material testing machine (WH-50, Weiheng). For the uniaxial tensile test sample, the sample was cut into a rectangular shape with a length of 60 mm, a width of 10 mm, a thickness of 5 mm, a tensile rate of 50 mm/min. For the compression test sample, the sample was prepared into a cylindrical shape with a diameter of 10 mm, a height of 15 mm, a compression rate of 5 mm/min, and compression to 90% compression strain.
